# Effect of the Chinese Herbal Medicine SS-1 on a Sjögren’s Syndrome-Like Disease in Mice

**DOI:** 10.3390/life11060530

**Published:** 2021-06-07

**Authors:** Po-Chang Wu, Shih-Chao Lin, Lauren Panny, Yu-Kang Chang, Chi-Chien Lin, Yu-Tang Tung, Hen-Hong Chang

**Affiliations:** 1 Ph.D. Program in Translational Medicine, National Chung Hsing University, Taichung 402, Taiwan; d10752@mail.cmuh.org.tw; 2 Rong Hsing Research Center for Translational Medicine, National Chung Hsing University, Taichung 402, Taiwan; 3 Rheumatology and Immunology Center, China Medical University Hospital, Taichung 408, Taiwan; 4 College of Medicine, China Medical University, Taichung 408, Taiwan; 5 Bachelor Degree Program in Marine Biotechnology, College of Life Sciences, National Taiwan Ocean University, Keelung 202301, Taiwan; sclin@mail.ntou.edu.tw; 6 Department of Biomedical Sciences and Pathobiology, Virginia-Maryland College of Veterinary Medicine, Virginia Polytechnic Institute and State University, Blacksburg, VA 24061, USA; laurenpanny@vt.edu; 7 Department of Medical Research, Tungs’ Taichung Metro Harbor Hospital, Taichung 433, Taiwan; t12193@ms.sltung.com.tw; 8 Department of Nursing, Jen-Teh Junior College of Medicine and Management, Miaoli 356, Taiwan; 9 The iEGG and Animal Biotechnology Center, Institute of Biomedical Science, National Chung-Hsing University, Taichung 402, Taiwan; 10 Department of Medical Research, China Medical University Hospital, Taichung 404, Taiwan; 11 Department of Medical Research, Taichung Veterans General Hospital, Taichung 407, Taiwan; 12 Department of Pharmacology, College of Medicine, Kaohsiung Medical University, Kaohsiung 807, Taiwan; 13 Graduate Institute of Biotechnology, National Chung Hsing University, Taichung 402, Taiwan; 14 Cell Physiology and Molecular Image Research Center, Wan Fang Hospital, Taipei Medical University, Taipei 116, Taiwan; 15 Graduate Institute of Integrated Medicine, and Graduate Institute of Acupuncture Science, College of Chinese Medicine, and Chinese Medicine Research Center, China Medical University, Taichung 408, Taiwan; 16 Department of Chinese Medicine, China Medical University Hospital, Taichung 408, Taiwan

**Keywords:** Sjögren’s syndrome, Chinese herbal medicine, SS-1, anti-inflammation, immunomodulation

## Abstract

Sjögren’s syndrome (SS) is an inflammatory autoimmune disease primarily affecting the exocrine glands; it has a major impact on patients’ lives. The Chinese herbal formula SS-1 is composed of Gan Lu Yin, Sang Ju Yin, and Xuefu Zhuyu decoction, which exerts anti-inflammatory, immunomodulatory, and antifibrotic effects. Our previous study demonstrated that SS-1 alleviates clinical SS. This study aimed to evaluate the efficacy and mechanism of the Chinese herbal formula SS-1 for salivary gland protein-induced experimental Sjögren’s syndrome (ESS). These results showed that ESS treatment with the Chinese herbal formula SS-1 (1500 mg/kg) significantly alleviated the severity of ESS. We found that SS-1 substantially improved saliva flow rates in SS mice and ameliorated lymphocytic infiltrations in submandibular glands. In addition, salivary gland protein-induced SS in mice treated with SS-1 significantly lowered proinflammatory cytokines (including IFN-γ, IL-6, and IL-17A) in mouse salivary glands and decreased serum anti-M3R autoantibody levels. In addition, we found that CD4+ T cells isolated from SS-1-treated SS mice significantly reduced the percentages of IFN-γ-producing CD4+ T cells (Th1) and IL-17A-producing CD4+ T cells (Th17). Our data show that SS-1 alleviates ESS through anti-inflammatory and immunomodulatory effects, which provides new insight into the clinical treatment of SS.

## 1. Introduction

Sjögren’s syndrome (SS) is an inflammatory autoimmune disease affecting primarily the exocrine glands [1]. Lymphocytic infiltrates destroy the functional epithelium, leading to decreased exocrine secretions (exocrinopathy) [2]. The hallmark symptoms of SS are dry mouth and dry eyes, while some patients may also present with various organ manifestations, such as interstitial lung disease, peripheral neuropathy, cutaneous vasculitis, and interstitial nephritis [3].

Currently, the therapeutic armamentarium for primary SS lacks a proven disease-modifying drug [4]. Corticosteroids and other immunosuppressive drugs are often prescribed for the treatment of organ-threatening extraglandular disease [5]; however, the clinical responses are often limited. Some patients seek Chinese herbal medicine (CHM) to relieve symptoms. However, to date, there is no scientific evidence that specific CHMs can effectively treat SS. For this purpose, we conducted a randomized, double-blind, placebo-controlled, clinical trial (Clinicaltrials.gov NCT02110446) and confirmed the therapeutic efficacy of the Chinese herbal formula SS-1 in patients with Sjögren’s syndrome. The Chinese herbal formula SS-1 is composed of Gan Lu Yin (GLY), Sang Ju Yin (SJY), and Xuefu Zhuyu decoction (XZD), which exert anti-inflammatory [6], immunomodulatory [7], and antifibrotic [8] effects, respectively. Recently, Lee et al. used murine T cell-based assays to investigate the effect of SS-1 on T cell responses [9]. Researchers have concluded that SS-1 inhibits T cell activation and abrogates T_H_ responses in Sjögren’s syndrome [9].

Recently, an experimentally induced auto-immunized mouse model of SS was established by Lin et al. [10]. Wild type C57BL/6 mice were immunized with submandibular gland (SG) proteins to induce experimental Sjögren’s syndrome (ESS). This auto-immunization-induced model recapitulates the key features of human SS and may have potential for studying the pathogenesis of human SS [11].

In this study, we examined the efficacy and mechanism of the Chinese herbal formula SS-1 in experimental Sjögren’s syndrome. We evaluated the effects of SS-1 on saliva amounts, cytokine production, histopathology of salivary glands, and serum autoantibodies in ESS.

## 2. Materials and Methods

### 2.1. SS-1 Formula

SS-1 comprises extracts of Gan Lu Yin (GLY), Sang Ju Yin (SJY), and Xuefu Zhuyu decoction (XZD) at a ratio of 2:1:1. The composition of the formula was described in detail previously [12] and is briefly summarized as follows. GLY comprises Ju-Hua (*Chrysanthemum morifolium* (Ramat.) Tzvel.), Gou-Qi (*Lycium barbarum*), Shou-Di-Huang (*Rehmannia glutinosa* Libosch.), Shan-Zhu-Yu (*Cornus officinalis* Sieb. Et Zucc.), Shan-Yao (*Dioscorea opposita* Thunb.), Ze-Xie (*Alisma orientalis* (Sam.) Juzep.), Fu-Ling (*Poria cocos* (Schw.) Wolff), and Mu-Dan-Pi (*Paeonia suffruticosa* Andr.), for which the therapeutic actions and indications are “nourish the liver and brighten the eyes” for antioxidant stress [13]. SJY comprises Sang-Ye (*Morus alba* L.), Ju-Hua (*C. morifolium* (Ramat.) Tzvel.), Lian-Qiao (*Forsythia suspense* (Thunb.) Vahl), Bo-He (*Mentha haplocalyx* Briq.), Jie-Geng (*Platycodon grandiflorum* (Jacq.) A. DC.), Zhi-Gan-Cao (*Glycyrrhiza glabra* L.), and Lu-Gen (*Phragmites communis* Trinus), for which the therapeutic actions and indications are “course wind and discharge heat” for anti-inflammation and immunomodulation [7,14]. XZD comprises Dang-Gui (*Angelica sinensis* (Oliv.) Diels), Sheng-Di-Huang (raw *R. glutinosa* Libosch.), Tao-Ren (*Prunus persica* (L.) Batsch), Hong-Hua (*Carthamus tinctorius* L.), Zhi- Ke (*Citrus aurantium* L.), Chi-Shao (red *Paeonia lactiflora* Pall.), Chai-Hu (*Bupleurum chinense* DC.), Zhi-Gan-Cao (*G. glabra* L.), Jie-Geng (*P. grandiflorum* (Jacq.) A. DC.), Chuan-Qiong (*Ligusticum chuanxiong* Hortorum), and Niu-Xi (*Achyranthes bidentata* Blume), for which the therapeutic actions and indications are “quicken the blood and dispel stasis” for immunomodulation and anti-fibrosis [15,16]. Kaiser Pharmaceuticals Co., Ltd. (Taiwan), a well-known pharmaceutical company that specializes in making traditional Chinese medicine (TCM), manufactured the powder and extract of SS-1 used in this study. All data, methods, locations, and collections of the experimental SS-1 samples were authenticated by licenses.

### 2.2. Mice

All animal experimental procedures followed published guidelines approved by the Institutional Animal Care and Use Committee (IACUC, NO.2020-155) of China Medical University. Eight-week-old female C57BL/6 mice (20–22 g weight) were purchased from the National Laboratory Animal Center (Taipei, Taiwan) and housed under specific pathogen-free (SPF) conditions. Mice were randomly divided into three groups (*n* = 5 in each group). One of the groups was fed water (naïve group) as control mice, and the other two groups were fed water and SS-1 as SS mice (SS-water and SS-SS1 groups, respectively). To investigate the effect of SS-1, the drug (1500 mg/kg bw) was administered orally twice a day from day 14 to 63 after the first immunization. As a control, the same quantity of water was administered.

### 2.3. Induction of SS Model

The SS model was induced by immunization with SG autoantigen as previously described with some modifications [10,17,18]. Five C57BL/6 mice were sacrificed under an overdose of pentobarbital. The bilateral SGs of the mice were immediately removed under sterile conditions, dissected free from the surrounding fat and connective tissues and weighed. The bilateral SG was homogenized in 2 mL of sterile saline solution per 100 mg of SG and then centrifuged at 3000 *g* for 15 min at 4 °C. The supernatant was collected, and the protein concentration of the supernatant was determined using the bicinchoninic acid (BCA) assay (Sigma-Aldrich, Buchs, Switzerland), and then adjusted to 800 μg of protein per 1 mL of PBS, and emulsified in an equal volume of complete Freund’s adjuvant (CFA, Sigma–Aldrich, St. Louis, MO, USA) to a concentration of 400 μg of protein per 1 mL of solution. On day 0, each of the mice was injected subcutaneously with 0.1 mL of the emulsion. On day 14, the booster injection was carried out with the same dose of autoantigen emulsified in Freund’s incomplete adjuvant (IFA, Sigma–Aldrich, St. Louis, MO, USA). Control mice (naïve group) were immunized with 0.1 mL of PBS per mouse on days 0 and 14.

### 2.4. Saliva Flow Rate Measurement

Saliva flow rates were measured as described in a previous study [11]. Briefly, on days 28, 42, and 63 after immunization, mice were anesthetized and injected intraperitoneally with pilocarpine (5 mg/kg bw). After pilocarpine injection for 10 min, saliva was collected immediately from the oral cavity using a 20-μL pipet tip.

### 2.5. Histology

On day 63, the SG was removed. Formaldehyde-fixed paraffin-embedded SGs were cut longitudinally to a thickness of 6 μm for hematoxylin and eosin staining. In addition, the sections were pretreated using heat-mediated antigen retrieval with EDTA buffer (pH 9, epitope retrieval solution) for 20 min. The sections were incubated in 0.02% hydrogen peroxide to block endogenous peroxidase activity and then incubated overnight at 4 °C with primary rabbit antibodies against mouse CD3+ and CD19+ in PBS with 0.1% BSA. The next day, the sections were immune-stained using HRP-conjugated goat anti-rabbit secondary antibody in PBS with 0.1% BSA for 30 min at room temperature. Diaminobenzidine was used for staining development, and the sections were counterstained with hematoxylin. Sections were stained and analyzed independently by two observers in a blind fashion. For the quantification of immune infiltrating cells, a light microscope with a 10× eyepiece and a 40× objective lens (400×) was used.

### 2.6. Quantitative Real-Time (RT)-PCR Analysis

Total RNA of SGs or cultured cells was extracted using TRIzol reagent (Life Technologies, Carlsbad, CA, USA) according to the manufacturer’s instructions and then reverse transcribed (RT) into cDNA using a Transcriptor First Strand (Roche Diagnostics, Germany) according to the manufacturer’s protocol. For further PCR amplification, an aliquot (1:10) of the RT product was adjusted to contain 0.1 μg of each primer, and additional buffer was added to a total volume of 20 μL. Real-time RT-PCR was performed using SYBR Green I FastStart Master Mix (Roche Molecular Diagnostics, Germany) with an Eco Real-Time PCR System (Illumina Inc. San Diego, CA. USA). To evaluate gene expression, real-time RT-PCR was performed with five target genes (TNF-α, IFN-γ, IL-6, IL-17A, and IL-4) using cDNA from SGs. β-actin GAPDH was used as an internal control.

### 2.7. Analysis of Anti-M3R and SSA IgG Antibody Production

Serum samples were collected on days 28, 42, and 63 after immunization. Serum levels of IgG against Sjögren’s syndrome-related antigen A (SSA) and M3 muscarinic receptor (M3R) were examined by a standard sandwich enzyme-linked immunosorbent assay (ELISA, PeproTech EC, London, UK). Briefly, 96-well MaxiSorp plates were coated with antigen peptides (5 μg/mL) at 4 °C overnight. Plates were washed and incubated with blocking buffer (0.5% gelatin, 0.5% bovine serum albumin and 0.05% Tween 20 in PBS) at room temperature for 1 h. Serum samples were diluted (1:100) and incubated in plates for 2 h at room temperature, followed by incubation with biotin-conjugated anti-mouse IgG (BioLegend, San Diego, CA, USA, 0.5 μg/mL) for 1 h. After washing, HRP streptavidin (BioLegend, San Diego, CA, USA, 1:1000) was added, and the plates were incubated for 30 min. Then, plates were washed, and freshly prepared TMB substrate (BioLegend, San Diego, CA, USA, 50 μL) was added. After 10 min, 20 μL of stop solution (3 M H_2_SO_4_) was added, and the absorbance at 450 nm was measured using a Sunrise microplate reader (Tecan, Männedorf, Switzerland). Antigenic peptides of SSA (AVALREYRKKMDIPA) and M3R (VLVNTFCDSCIPKTYWNLGY) were synthesized chemically by a solid-phase approach and purified by high-performance liquid chromatography (SBS Genetech Co., Ltd., Beijing, China).

### 2.8. Cell Proliferation and Cytokine Production Analysis

At day 63, spleens were isolated and stimulated with homogenized SG autoantigen (50 μg/mL) in a flat-bottom 96-well plate (Corning Inc. Corning, NY, USA) for 96 h, and then the cells were pulsed with [3H] thymidine for 18 h before harvesting. The incorporation of radioactivity was measured in a beta-counter (Beckman Instruments). The IFN-γ and IL-17A production in the cell supernatants was determined by ELISA.

For intracellular detection of cytokines, mouse spleen cells were incubated with SG autoantigen for 48 h. GolgiStop (BD Biosciences, San Diego, CA, USA) solution was added 6 h before harvesting the cultured cells. The cells were then washed twice in FACS buffer and stained with phycoerythrin-conjugated anti-mouse CD4 (BioLegend, San Diego, CA, USA). Splenocytes were fixed and subjected to intracellular staining using the Cytofix/Cytoperm Plus Kit (BD Biosciences, San Diego, CA, USA) according to the manufacturer’s instructions (BD Biosciences, San Diego, CA, USA). FITC-conjugated mAbs specific to murine IFN-γ and IL-17A were purchased from BioLegend (San Diego, CA, USA). All samples were detected on an Accuri C5 cytometer using C6 Accuri system software (Accuri Cytometers Inc., Ann Arbor, MI, USA).

### 2.9. Assay of Dendritic Cell (DC) Maturation in Vivo

On day 63, the single-cell suspension from the spleen was pressed through sterile stainless steel mesh. After lysis of red blood cells using commercial RBC lysis buffer (Sigma-Aldrich), the cells were washed several times with RPMI 1640 medium and stained with mAbs specific for mouse CD11C, CD80, and CD86 (BioLegend, San Diego, CA, USA), as well as control isotype-matched antibody. Stained cells were subjected to an Accuri 5 flow cytometer (BD Bioscience, San Jose, CA, USA), and the mean fluorescence intensity was calculated using C6 Accuri system software (BD Bioscience, San Jose, CA, USA). The cytokine mRNA expression levels of IL-12, IL-23, and IL-4 were examined using real-time PCR.

### 2.10. Assay of Bone Marrow Derived DC Maturation in Vitro

Immature DCs were cultured in the presence or absence of SS-1 extract dilution (1:1000; 1:2000) for 1 h followed by stimulation with lipopolysaccharide (LPS, 100 ng/mL) for 16 h and stained with mAbs specific for mouse CD11C, CD80, and CD86 (BioLegend, San Diego, CA, USA). After mAb staining, the samples were detected on an Accuri 5 flow cytometer (BD Bioscience, San Jose, CA, USA), and the mean fluorescence intensity was calculated using C6 Accuri system software (BD Bioscience, San Jose, CA, USA). The cytokines IL-12 and IL-23 in culture supernatants were analyzed using ELISA kits (PeproTech, London, UK).

### 2.11. Statistical Analysis

Data are expressed as the mean ± standard deviation (*n* = 5). One-way ANOVA with a post hoc Dunnett test was used to compare multiple experimental groups with GraphPad Prism v5.0 software (La Jolla, CA, USA). A *p*-value of less than 0.05 was considered a significant difference.

## 3. Result

### 3.1. The Effect of SS-1 on SS-like Symptoms in a Salivary Gland-Induced SS Mouse Model

**Figure 1:**
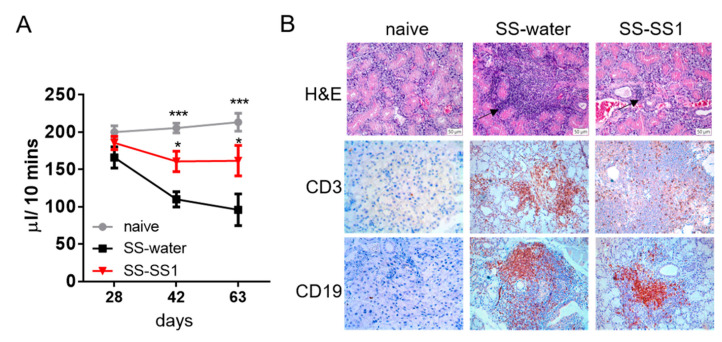
SS-1 improved saliva flow rates in SS mice and ameliorated lymphocytic infiltrations in submandibular glands. (**A**) Saliva flow rates of mice at days 28, 42, and 63 after SS-1 treatment. Data are means ± SEMs (*n* = 5). The data presented are representative of three independent experiments with similar results. * *p* < 0.05 and *** *p* < 0.001 versus SS-water group. (**B**) Representative histological patterns of submandibular glands determined by hematoxylin and eosin (H&E) staining and immunostaining with anti-mouse CD3 antibody or anti-mouse CD19 antibody, original magnification ×100.

We used a mouse model of SS-like symptoms induced by salivary glands to evaluate the therapeutic effect of SS-1 on the progression of SS. The results showed that the saliva flow rate in the SS-1-treated group was significantly increased compared to that in the water-treated group (Figure 1A). Compared to water-treated mice, the lymphocytic infiltration, T cells (CD3+) and B cells (CD19+) in the submandibular glands of the SS-1-treated group were decreased (Figure 1B).

### 3.2. The Effect of SS-1 on the Production of Inflammatory Cytokines in a Mouse Salivary Gland-induced SS Mouse Model

**Figure 2:**
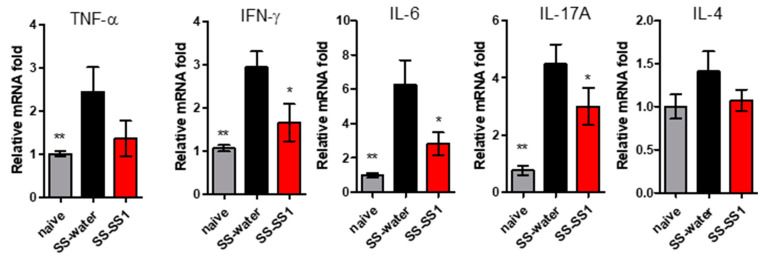
RNA expression of TNF-α, IFN-γ, IL-6, IL-17A, and IL-4 in salivary gland homogenates. These inflammatory cytokines were measured by qRT-PCR. The data were normalized to GAPDH expression in each sample. Data are means ± SEMs (*n* = 5). The data presented are representative of three independent experiments with similar results. * *p* < 0.05 and ** *p* < 0.01 versus SS-water group.

Excessive production of proinflammatory cytokines is one of the important pathological indications of SS. Therefore, we investigated the effect of SS-1 on the production of proinflammatory cytokines. On day 63, the salivary gland was removed and homogenized at the end of the experiment. The mRNA levels of proinflammatory cytokines (TNF-α, IFN-γ, IL-6, IL-17A, and IL-4) were determined by qRT-PCR. As shown in Figure 2, the TNF-α, IFN-γ, IL-6, and IL-17A levels of SS mice were significantly increased relative to those of the naïve group. However, the levels of proinflammatory cytokines (including IFN-γ, IL-6, and IL-17A) in the salivary glands of SG immunization mice after SS-1 treatment were significantly reduced.

### 3.3. The Effect of SS-1 on Anti-SSA and Anti-M3R Antibodies in a SG-Induced SS Mouse Model

**Figure 3:**
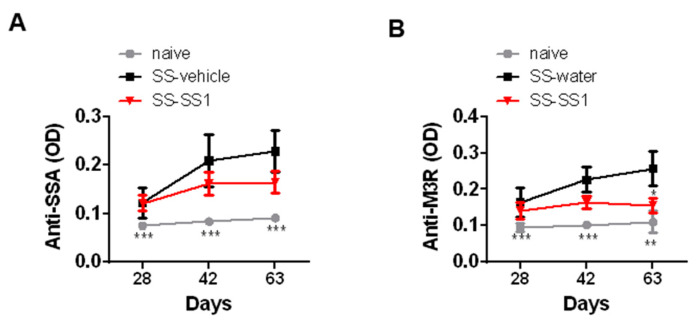
SS-1 decreased M3R-specific IgG antibody production in the serum of SS mice. The presence of anti-SSA and anti-M3R antibodies in the serum of mice collected at days 21, 42, and 63 was detected by ELISA. Data are means ± SEMs (*n* = 5). The data presented are representative of three independent experiments with similar results. * *p* < 0.05, ** *p* < 0.01 and *** *p* < 0.001 versus SS-water group.

The pathogenic mechanisms of SS involve the synergistic actions of anti-SSA and anti-M3R antibodies. The results showed a significant increase in SSA and M3R-specific IgG in the serum collected on days 28, 42, and 63 in all SS mice compared with naïve mice (Figure 3). However, SS-1 treatment significantly reduced the level of anti-M3R IgG (Figure 3) on day 63 but had no significant effect on anti-SSA.

### 3.4. The Effect of SS-1 on Th1 and Th17 Cell Expansion in the Spleen

**Figure 4:**
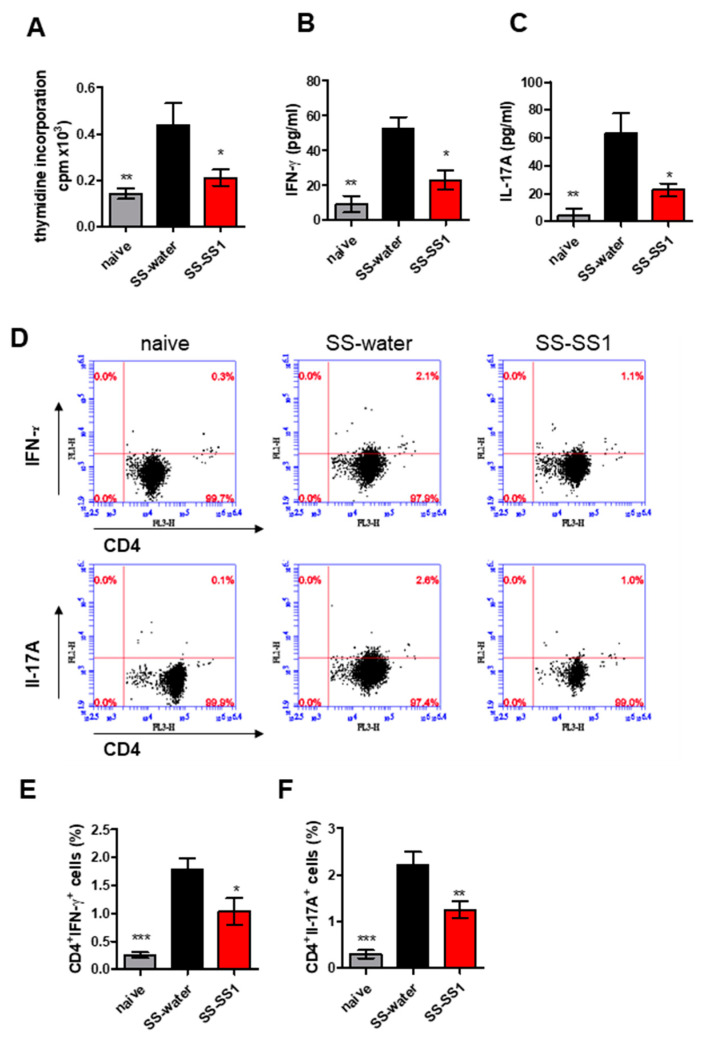
SS-1 inhibited salivary gland extract-induced specific T cell proliferation and IFN-γ and IL-17A expression in splenic CD4+ T cells from SS mice. (**A**) Spleens were harvested on day 63 and cultured in the presence of salivary gland extracts for 96 h. After pulsing with 1 μCi of [3H]thymidine per well for the last 18 h, proliferation was determined as radioactivity incorporation in counts per minute (cpm). Regarding the in vitro cytokine analysis, the culture supernatants were collected, and (**B**) IFN-γ and (**C**) IL-17A were measured by ELISA. (**D**) Representative flow cytometric dot plots of intracellular staining of IFN-γ and IL-17A in CD4+ T cells for each group are shown. Bar graphs of (**E**) IFN-γ and (**F**) IL-17A in CD4+ T cells represent the mean ± SEM (*n* = 5) from three independent experiments. * *p* < 0.05, ** *p* < 0.01 and *** *p* < 0.001 versus SS-water group.

To study the effect of SS-1 on salivary gland protein-specific T cell responses, spleen cells were isolated from different experimental groups and incubated with salivary gland extract for 72 h in vitro (Figure 4). Compared with cells isolated from the water-treated group, salivary gland extract isolated from the SS-1-treated group significantly decreased the stimulation of cell proliferation (Figure 4A). In addition, compared with the vehicle control mice, the levels of IFN-γ and IL-17A in the supernatants of the lymphocytes were significantly lower in the SS-1-treated mice (Figure 4B,C). To further verify whether SS-1 can modulate populations of different T cells during SS, the distribution of CD4+ T cell subsets in spleen cells was analyzed using flow cytometry. After stimulation with salivary gland extract, flow cytometric analysis showed that CD4+ T cells isolated from SS-1-treated SS mice significantly lowered the numbers of CD4+ IFN-γ+ Th1 and CD4+ IL-17A+ Th17 cells compared with water-treated SS mice (Figure 4D–F).

### 3.5. The Effect of SS-1 on Splenic DC Maturation In Vivo

**Figure 5:**
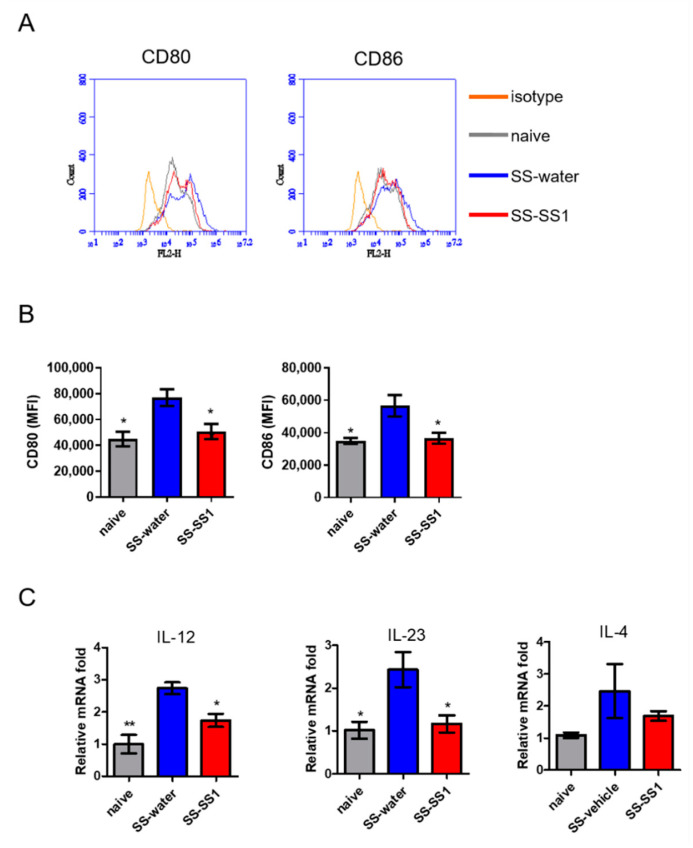
SS-1 inhibited the expression of costimulatory molecules and cytokines on spleen DCs. (**A**) The expression of CD80 and CD86 on spleen DCs was analyzed by flow cytometry. (**B**) The expression levels of CD80 and CD86 are presented as the mean fluorescence intensity (MFI). (**C**) IL-12, IL-23, and IL-4 mRNA was detected in purified CD11C+ DCs from the spleen using quantitative real-time PCR. The data were normalized to HPRT expression in each sample and the mean ± SEM (*n* = 5). * *p* < 0.05 and ** *p* < 0.01 versus SS-water group.

Owing to the extensive capability of DCs to activate naïve T cells, we further evaluated the effect of SS-1 on DC maturation *in vivo*. On day 63, splenic DCs were purified and analyzed for phenotypic maturation by flow cytometry. As shown in Figure 5A,B, SS-1-treated mice significantly reduced CD80 and CD86 (maturation markers) expression on gated CD11C+ splenic cells compared with vehicle control mice. Consistent with the activated phenotype, DCs from the spleens of the SS-1-treated mice produced lower levels of IL-12 and IL-23, which play critical roles in the induction of Th1 and Th17 immune responses, than those from the vehicle control mice (Figure 5C), but there was no difference in the levels of Th2 cytokine (IL-4) among all groups.

### 3.6. The Effect of SS-1 on Co-Stimulatory Molecules and Cytokine Production in Murine Bone Marrow-Derived Dendritic Cells (BMDCs) Induced by LPS

**Figure 6:**
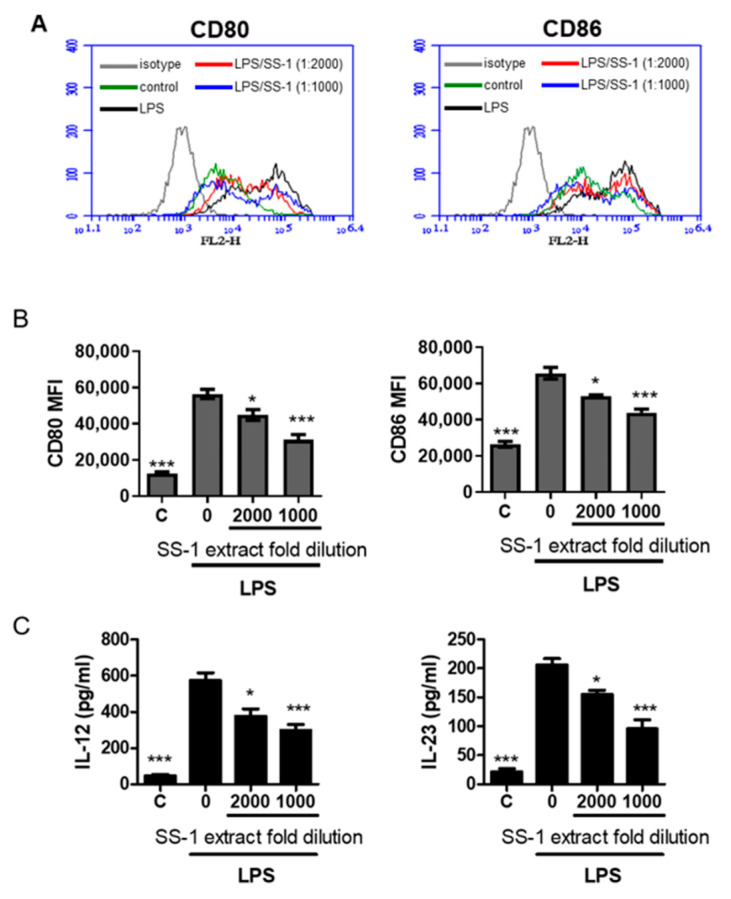
SS-1 reduced LPS-induced surface costimulatory markers and cytokine production in BMDCs. (**A**) BMDCs from naïve mice were stimulated with LPS and treated with or without SS-1 at the indicated concentrations followed by evaluation of costimulatory marker expression (CD80 and CD86). (**B**) The mean fluorescence intensity (MFI) of cellular marker molecule expression (CD80 and CD86) was quantified after gating on CD11c+ cells. (**C**) IL-12 and IL-23 cytokine production was detected by ELISA. Data are means ± SEMs (*n* = 5). The data presented are representative of three independent experiments with similar results. * *p* < 0.05, *** *p* < 0.001 versus LPS + water-treated control.

Following the suppression of splenic DC maturation in Figure 5, we attempted to characterize more changes in BMDCs affected by SS-1 in vitro. We activated and treated immature BMDCs from naïve mice with LPS and SS-1 and then measured the expression of costimulatory molecules. The expression levels of the costimulatory markers CD80 and CD86, both of which enable DCs to prime downstream T cell differentiation, were detected. Figure 6A,B show that SS-1 significantly reduced CD80 and CD86 under both treatment concentrations. DCs can produce inflammatory cytokines, and these cytokines can guide the differentiation of naïve T cells [5,6]. Therefore, the DC cytokine profile stimulated by LPS followed by treatment with or without SS-1 was evaluated. The results showed that SS-1 treatment remarkably reduced Th1-biased cytokines (IL-12) and Th17-biased cytokines (IL-23) compared with the levels in water-treated SS mice (Figure 6C).

## 4. Discussions

In this study, we investigated the efficacy and mechanisms of the Chinese herbal formula SS-1 in experimental Sjögren’s syndrome. In SS, the salivary glands are infiltrated and destroyed by inflammatory cells, which further produce cytokines to enhance the damage of glandular epithelium, resulting in reduced exocrine function and increased autoantibody level [19,20]. In addition, damaged epithelial cells induce the release of inflammatory factors by which the overactive immune response will be aggravated [20]. As a result, the destruction of exocrine glands, featured by lower saliva flow rate in SS, is highly related to the continuous infiltration of lymphocytes [21]. Our data supports the clinical signs of SS, such as saliva flow rate, could be improved by SS-1 treatment (Figure 1A) in SS mice. It also confirms decreases in infiltrations of T (CD3) and B (CD19) lymphocyte in SGs, as well as the expression of inflammatory factors, including IFN-γ, IL-6, and IL-17A, in mouse salivary glands leads to the alleviation of SS symptoms (Figure 2). Moreover, the serum anti-M3R autoantibody levels decreased. This therapeutic effect may be related to the ability of SS-1 to inhibit dendritic cell maturation, hence suppressing the generation of Th1 and Th17 cells.

Most of the infiltrating cells are CD4+ T-cells and, to a lesser degree, antibody-secreting B cells [22]. T cells that secrete IFN-γ and IL-17 have been detected in inflamed salivary glands of patients with SS, suggesting a predominantly Th1- and Th17-driven response in the pathogenesis of SS [23]. Dendritic cells are also found in proximity to the ductal epithelium, where they secrete inflammatory cytokines such as TNF-α, IL-6, and IL-12 [24]. TNF-α, cooperating with IFN-γ, induces apoptosis of salivary gland cells [25]. IL-12 promotes the differentiation of IFN-γ-producing T cells [25], while IL-6 participates in the generation of Th17 cells and fosters their proliferation [26]. In the presence of IL-6, Th17 cells also orchestrate the development of germinal centers (GCs) dominated by autoreactive lymphocytes [27]. These GC-like structures provide an opportune environment for the differentiation of antigen-driven B cell responses in the salivary glands [28]. Anti-SSA/Ro and anti-La/SSB are the hallmark autoantibodies in SS, which play an important role in disease diagnosis. Autoantibodies against muscarinic acetylcholine receptor M3 (M3R) have also been shown to exist in the sera of SS patients [29] and may have a role in causing glandular dysfunction [30].

Recently, an experimental SS mouse model was established by Lin et al. [11]. Upon immunization with autoantigenic peptides derived from salivary glands, the mice displayed decreased saliva production, increased anti-SSA and anti-M3R autoantibodies, and extensive glandular inflammation with the infiltration of both T and B cells, which recapitulates the key features of human SS [10]. In this ESS model, Th17 cells also play a critical role in disease pathogenesis [11]. Hence, several studies have been carried out to investigate potential therapeutic drugs and molecular mechanisms against SS [31,32,33].

At present, no disease-modifying therapies are approved to treat SS [4]. Oral glucocorticoids and immunosuppressive medications, including hydroxychloroquine, azathioprine, cyclosporine, and methotrexate, are often prescribed; however, they have not been found to improve glandular function in clinical trials [34,35]. Some patients seek traditional Chinese medicines (TCMs) to relieve symptoms. Several TCMs have been reported to be beneficial in treating autoimmune diseases [36]. In our previous network analysis, we identified the core pattern prescription for treating SS [12]. This core pattern prescription contains GLY, which exerts anti-inflammatory properties [6]. An in vitro human oral cancer cell line study demonstrated that GLY suppresses TNF-α secretion through NF-κB-, AKT-, and ERK-dependent pathways [6]. SJY is among the most commonly prescribed Chinese herbal formulas for viral infections. One prospective cohort study suggests that SJY might have immunomodulatory effects, including a decrease in the number of B-lymphocytes [37]. XZD is a common herbal formula that has been used to treat blood stasis syndrome. Animal studies demonstrate that XZD has antifibrotic properties [8]. Since tissue fibrosis is a common consequence of chronic inflammation, and salivary gland fibrosis in SS is elevated [38], we incorporated XZD in our innovative herbal combination, SS-1. The herbal formula SS-1 is composed of GLY, SJY, and XZD. This combination is created to allow good general acceptance to patients with different disease states. Moreover, we conducted a randomized, double-blind, placebo-controlled, clinical trial (Clinicaltrials.gov NCT02110446) and confirmed the therapeutic efficacy of the Chinese herbal formula SS-1 in patients with Sjögren’s syndrome. Significant relief of sicca symptoms, as well as downregulation of lymphocyte activities, were noted in our clinical trial.

T cells are known to play an important role in SS [39]. Our previous study pointed out that SS-1 inhibits Th1 (CD4+ IFN-γ+ and CD4+ TNF-α+ cells) and Th2 (CD4+ IL-4+ c cells) polarization of mouse spleen cells and T cell proliferation in Sjögren’s syndrome [9]. In this study, we also found that SS-1 inhibited splenic T cell proliferation (Figure 4A), Th1 (CD4+ IFN-γ+) and Th17 (CD4+ IL-17+) cells (Figure 4C,D), and reduced the levels of cytokines (IFN-γ and IL-17) in a salivary gland-induced SS-like symptom mouse model (Figure 4B).

Vogelsang et al. [40] also proposed the possible role of DCs in SS. However, previous studies lacked the regulatory mechanism of SS-1 with T cells and DCs. Therefore, we reviewed previous studies of GLY, SJY, and XZD. GLY exerts anti-inflammatory and ameliorating effects on systemic lupus erythematosus [41]. Inagaki et al. pointed out that a concentration of more than 0.05 mg/mL GLY significantly inhibited sRANKL-induced osteoclast differentiation in RAW264.7 cells, thereby inhibiting bone resorption in an experimental periodontitis model. GLY has a significant protective effect in traditional Chinese medicine formulations commonly used in systemic lupus erythematosus patients [42]. SJY has beneficial immunomodulatory effects for preventing viral infections [7]. Healthy people, after taking two herbal formulas (SJY and Yu Ping Feng San), have a short-term increase in their CD4/CD8 ratio, so herbal formulas can prevent viral infections, including SARS [7]. In addition, a previous study showed that XZD could act as an anti-inflammatory drug in a rat model of traumatic brain injury by inhibiting the PI3K-AKT-mTOR pathway [43]. Xing et al. also proposed that XZD has neuroprotection in a rat model of traumatic brain injury through the neutralization of proinflammatory cytokines (such as TNF-α and IL-1β). These findings support our findings that SS-1 can inhibit the production of proinflammatory cytokines (TNF-α and IL-6) in SS mice.

A previous study showed that SS patients have higher IL-12 mRNA produced by DCs and IL-12 expression in serum than healthy people. However, IL-12 levels in SS patients were not related to anti-SSA or anti-SSB antibodies [44]. During the occurrence of SS-like symptoms in non-obese diabetic (NOD) mice, mesenchymal stem cells (MSCTs) enhanced salivary flow rates and decreased lymphocyte infiltrations in the salivary glands of NOD mice and downregulated Th17 and Tfh cells, but upregulated regulatory T cells [44]. However, they had no effect on plasma cells, Th1, and Th2 cells [41]. The results of our study on splenic DCs and BMDCs of SS-1-treated mice produced lower levels of IL-12 and IL-23 than vehicle control mice, thereby reducing Th1 (CD4 + IFN-γ) and Th17 (CD4 + IL-17) cells.

Recent data suggest that glandular hypofunction in SS could result from functional suppression by anti-M3R autoantibodies [27,45,46]. SS-1 treatment significantly decreased the levels of anti-M3R IgG (Figure 3); hence, improving the secretory function of the salivary glands. Nevertheless, SS-1 treatment had no significant effect on the levels of anti-SSA. This result is compatible with findings from previous studies showing that the titers of anti-Ro/SSA antibodies do not change after treatment [47,48,49]. Even when B cell depletion therapy with rituximab is used, the levels of anti-Ro/SSA antibodies remain constant [47,50].

## 5. Conclusions

SS-1 treatment leads to beneficial therapeutic effects by improving saliva flow rates and lymphocytic infiltration in SG protein-induced ESS due to the regulation of several inflammatory mediators. These mediators include Th1-mediated cytokines and Th17-mediated cytokines. These results indicate that SS-1 treatment may be an effective and safe therapeutic strategy and can be used as an immunomodulator for T cell-mediated autoimmune diseases, including SS.

## Data Availability

The data that support the findings of this study are available from the corresponding author upon reasonable request.

## References

[1] Fox R.I. (2005). Sjogren’s syndrome. Lancet.

[2] Christodoulou M.I., Kapsogeorgou E.K., Moutsopoulos H.M. (2010). Characteristics of the minor salivary gland infiltrates in Sjogren’s syndrome. J. Autoimmun..

[3] Asmussen K., Andersen V., Bendixen G., Schiodt M., Oxholm P. (1996). A new model for classification of disease manifestations in primary Sjogren’s syndrome: Evaluation in a retrospective long-term study. J. Intern. Med..

[4] Leverenz D.L., St Clair E.W. (2019). Recent advances in the search for a targeted immunomodulatory therapy for primary Sjögren’s syndrome. F1000Research.

[5] Peri Y., Agmon-Levin N., Theodor E., Shoenfeld Y. (2012). Sjogren’s syndrome, the old and the new. Best Pract. Res. Clin. Rheumatol..

[6] Yang J.S., Wu C.C., Lee H.Z., Hsieh W.T., Tang F.Y., Bau D.T., Lai K.C., Lien J.C., Chung J.G. (2016). Suppression of the TNF-alpha level is mediated by Gan-Lu-Yin (traditional chinese medicine) in human oral cancer cells through the Nf-Kappa B, Akt, and Erk-dependent pathways. Environ. Toxicol..

[7] Poon P.M., Wong C.K., Fung K.P., Fong C.Y., Wong E.L., Lau J.T., Leung P.C., Tsui S.K., Wan D.C., Waye M.M. (2006). Immunomodulatory effects of a traditional chinese medicine with potential antiviral activity: A self–control study. Am. J. Chin. Med..

[8] Zhang G., Yang G., Deng Y., Zhao X., Yang Y., Rao J., Wang W., Liu X., He J., Lv L. (2016). Ameliorative effects of Xue-Fu-Zhu-Yu decoction, Tian-Ma-Gou-Teng-Yin and Wen-Dan decoction on myocardial fibrosis in a hypertensive rat mode. BMC Complement. Altern. Med..

[9] Lee G.A., Chang C.M., Wu Y.C., Ma R.Y., Chen C.Y., Hsue Y.T., Liao N.S., Chang H.H. (2021). Chinese herbal Mmedicine SS-1 inhibits T cell activation and abrogates Th responses in Sjogren’s syndrome. J. Formos. Med. Assoc..

[10] Lin X., Song J.X., Shaw P.C., Ng T.B., Wong R.N., Sze S.C., Tong Y., Lee K.F., Zhang K.Y. (2011). An autoimmunized mouse model recapitulates key features in the pathogenesis of Sjogren’s syndrome. Int. Immunol..

[11] Lin X., Rui K., Deng J., Tian J., Wang X., Wang S., Ko K.H., Jiao Z., Chan V.S., Lau C.S. (2015). Th17 cells play a critical role in the development of experimental Sjogren’s syndrome. Ann. Rheum. Dis..

[12] Chang C.M., Chu H.T., Wei Y.H., Chen F.P., Wang S., Wu P.C., Yen H.R., Chen T.J., Chang H.H. (2015). The core pattern analysis on chinese herbal medicine for Sjogren’s syndrome: A nationwide population–based study. Sci. Rep..

[13] Lin I.H. (2000). Mucositis and dry mouth due to NPC radiotherapy treated by chinese herb Gan-Lu-Yin. Yearb. Chin. Med. Pharm..

[14] Fang Z., Zhang M., Yi Z., Wen C., Qian M., Shi T. (2013). Replacements of rare herbs and simplifications of traditional chinese medicine formulae based on attribute similarities and pathway enrichment analysis. Evid. Based Complement. Alternat. Med..

[15] Shao S.J. (2008). The clinical application and research progress of Xie-Fu-Zhu-Yu-Tang. Beijing J. Tradit. Chin. Med..

[16] Ji C.Z., Zhang P.Y., Wang Y.X., Zhang D.S., Zhang L. (1999). Effect of Xuefuzhuyu soup on the levels of IL-2 and SIL-2R in mice serum. J. Qiqihar Med..

[17] White S.C., Casarett G.W. (1974). Induction of experimental autoallergic sialadenitis. J. Immunol..

[18] Wang Y., Yan T., Shen J., Guo H., Xiang X. (2007). Preventive effect of Ophiopogon japonicus polysaccharides on an autoallergic mouse model for Sjogren’s syndrome by regulating the Th1/Th2 cytokine imbalance. J. Ethnopharmacol..

[19] Campisi G., Paderni C., Saccone R., Fede O., Wolff A., Giannola L.I. (2010). Human buccal mucosa as an innovative site of drug delivery. Curr. Pharm. Des..

[20] Manoussakis M.N., Kapsogeorgou E.K. (2010). The role of intrinsic epithelial activation in the pathogenesis of Sjögren’s syndrome. J. Autoimmun..

[21] Shi B., Qi J., Feng R., Zhang Z., Chen W., Li W., Tang X., Yao G., Sun L. (2016). THU0280 IL-12 exacerbates Sjogren’s syndrome through inducing lymphocyte infiltrations into salivary glands and imbalance of lymphocyte subsets. Ann. Rheum. Dis..

[22] Fox R.I. (1996). Sjogren’s syndrome: Immunobiology of exocrine gland dysfunction. Adv. Dent. Res..

[23] Brito-Zeron P., Baldini C., Bootsma H., Bowman S.J., Jonsson R., Mariette X., Sivils K., Theander E., Tzioufas A., Ramos-Casals M. (2016). Sjögren syndrome. Nat. Rev. Dis. Primers.

[24] (2020). Firestein and Kelley’s Textbook of Rheumatology.

[25] Jin J.O., Yu Q. (2013). T cell-associated cytokines in the pathogenesis of Sjogren’s syndrome. J. Clin. Cell Immunol..

[26] Youinou P., Pers J.O. (2011). Disturbance of cytokine networks in Sjogren’s syndrome. Arthritis Res. Ther..

[27] Hsu H.C., Yang P., Wang J., Wu Q., Myers R., Chen J., Yi J., Guentert T., Tousson A., Stanus A.L. (2008). Interleukin 17-producing T helper cells and interleukin 17 orchestrate autoreactive germinal center development in autoimmune Bxd2 mice. Nat. Immunol..

[28] Hansen A., Lipsky P.E., Dörner T. (2007). B cells in Sjögren’s syndrome: Indications for disturbed selection and differentiation in ectopic lymphoid tissue. Arthritis Res Ther..

[29] Gao J., Cha S., Jonsson R., Opalko J., Peck A.B. (2004). Detection of anti–type 3 muscarinic acetylcholine receptor autoantibodies in the sera of Sjogren’s syndrome patients by use of a transfected cell line assay. Arthritis Rheum..

[30] Dawson L.J., Stanbury J., Venn N., Hasdimir B., Rogers S.N., Smith P.M. (2006). Antimuscarinic antibodies in primary Sjogren’s syndrome reversibly inhibit the mechanism of fluid secretion by human submandibular salivary acinar cells. Arthritis Rheum..

[31] Li H., Sun X., Zhang J., Sun Y., Huo R., Li H., Zhai T., Shen B., Zhang M., Li N. (2016). Paeoniflorin ameliorates symptoms of experimental Sjogren’s syndrome associated with down-regulating Cyr61 expression. Int. Immunopharmacol..

[32] Wu H., Chen X., Gu F., Zhang P., Xu S., Liu Q., Zhang Q., Wang X., Wang C., Korner H. (2020). Cp-25 alleviates antigen-induced experimental Sjogren’s syndrome in mice by inhibiting Jak1-Stat1/2-Cxcl13 signaling and interfering with B-cell migration. Lab. Investig..

[33] Huang Y., Yang G., Fei J., Wu Y., Yan J. (2020). Bufotalin ameliorates experimental Sjogren’s syndrome development by inhibiting Th17 generation. Naunyn Schmiedebergs Arch. Pharmacol..

[34] Pijpe J., Kalk W.W., Bootsma H., Spijkervet F.K., Kallenberg C.G., Vissink A. (2007). Progression of salivary gland dysfunction in patients with Sjogren’s syndrome. Ann. Rheum. Dis..

[35] Van der Heijden E.H.M., Kruize A.A., Radstake T.R.D.J., van Roon J.A.G. (2018). Optimizing conventional dmard therapy for Sjogren’s syndrome. Autoimmun. Rev..

[36] Ma H.D., Deng Y.R., Tian Z., Lian Z.X. (2013). Traditional chinese medicine and immune regulation. Clin. Rev. Allergy Immunol..

[37] Lau T.F., Leung P.C., Wong E.L., Fong C., Cheng K.F., Zhang S.C., Lam C.W., Wong V., Choy K.M., Ko W.M. (2005). Using herbal medicine as a means of prevention experience during the SARS crisis. Am. J. Chin. Med..

[38] Leehan K.M., Pezant N.P., Rasmussen A., Grundahl K., Moore J.S., Radfar L., Lewis D.M., Stone D.U., Lessard C.J., Rhodus N.L. (2018). Minor salivary gland fibrosis in Sjogren’s syndrome is elevated, associated with focus score and not solely a consequence of aging. Clin. Exp. Rheumatol..

[39] Yao Y., Ma J.F., Chang C., Xu T., Gao C.Y., Gershwin M.E., Lian Z.X. (2021). Immunobiology of T cells in Sjogren’s syndrome. Clin. Rev. Allergy Immunol..

[40] Vogelsang P., Jonsson M.V., Dalvin S.T., Appel S. (2006). Role of dendritic cells in Sjogren’s syndrome. Scand. J. Immunol..

[41] Inagaki Y., Kido J.I., Nishikawa Y., Kido R., Sakamoto E., Bando M., Naruishi K., Nagata T., Yumoto H. (2021). Gan-Lu-Yin (Kanroin), traditional chinese herbal extracts, reduces osteoclast differentiation in vitro and prevents alveolar bone resorption in rat experimental periodontitis. J. Clin. Med..

[42] Ma Y.C., Lin C.C., Li C.I., Chiang J.H., Li T.C., Lin J.G. (2016). Traditional chinese medicine therapy improves the survival of systemic lupus erythematosus patients. Semin. Arthritis Rheum..

[43] Xing Z., Xia Z., Peng W., Li J., Zhang C., Fu C., Tang T., Luo J., Zou Y., Fan R. (2016). Xuefu Zhuyu decoction, a traditional chinese medicine, provides neuroprotection in a rat model of traumatic brain injury via an anti-inflammatory pathway. Sci. Rep..

[44] Shi B., Qi J., Yao G., Feng R., Zhang Z., Wang D., Chen C., Tang X., Lu L., Chen W. (2018). Mesenchymal stem cell transplantation ameliorates Sjogren’s syndrome via suppressing Il-12 production by dendritic cells. Stem. Cell Res. Ther..

[45] Dawson L.J., Fox P.C., Smith P.M. (2006). Sjogrens Syndrome the non apoptotic model of glandular hypofunction. Rheumatology.

[46] Fayyaz A., Kurien B.T., Scofield R.H. (2016). Autoantibodies in Sjogren’s syndrome. Rheum. Dis. Clin. N. Am..

[47] Hernández-Molina G., Leal-Alegre G., Michel-Peregrina M. (2011). The meaning of anti-ro and anti-la antibodies in primary Sjogren’s syndrome. Autoimmun. Rev..

[48] Davidson B.K., Kelly C.A., Griffiths I.D. (1999). Primary Sjogren’s syndrome in the north east of england: A long term follow up study. Rheumatology.

[49] Praprotnik S., Bozic B., Kveder T., Rozman B. (1999). Fluctuation of anti-ro/SS-a antibody levels in patients with systemic lupus erythematosus and Sjogren’s syndrome: A prospective study. Clin. Exp. Rheumatol..

[50] Seror R., Sordet C., Guillevin L., Hachulla E., Masson C., Ittah M., Candon S., Guern V.L., Aouba A., Sibilia J. (2007). Tolerance and efficacy of rituximab and changes in serum B cell biomarkers in patients with systemic complications of primary Sjogren’s syndrome. Ann. Rheum. Dis..

